# Developing and Optimising the Use of Logic Models in Systematic Reviews: Exploring Practice and Good Practice in the Use of Programme Theory in Reviews

**DOI:** 10.1371/journal.pone.0142187

**Published:** 2015-11-17

**Authors:** Dylan Kneale, James Thomas, Katherine Harris

**Affiliations:** 1 Evidence for Policy and Practice Information and Co-ordinating Centre (EPPI-Centre), UCL Institute of Education, University College London, London, United Kingdom; 2 Centre for Paediatrics, Blizard Institute, Queen Mary University of London, London, United Kingdom; University of Toronto Dalla Lana School of Public Health, CANADA

## Abstract

**Background:**

Logic models are becoming an increasingly common feature of systematic reviews, as is the use of programme theory more generally in systematic reviewing. Logic models offer a framework to help reviewers to ‘think’ conceptually at various points during the review, and can be a useful tool in defining study inclusion and exclusion criteria, guiding the search strategy, identifying relevant outcomes, identifying mediating and moderating factors, and communicating review findings.

**Methods and Findings:**

In this paper we critique the use of logic models in systematic reviews and protocols drawn from two databases representing reviews of health interventions and international development interventions. Programme theory featured only in a minority of the reviews and protocols included. Despite drawing from different disciplinary traditions, reviews and protocols from both sources shared several limitations in their use of logic models and theories of change, and these were used almost unanimously to solely depict pictorially the way in which the intervention worked. Logic models and theories of change were consequently rarely used to communicate the findings of the review.

**Conclusions:**

Logic models have the potential to be an aid integral throughout the systematic reviewing process. The absence of good practice around their use and development may be one reason for the apparent limited utility of logic models in many existing systematic reviews. These concerns are addressed in the second half of this paper, where we offer a set of principles in the use of logic models and an example of how we constructed a logic model for a review of school-based asthma interventions.

## Introduction

Researchers in academic institutions have historically measured their success by the impact that their research has within their own research communities, and have paid less attention to measuring its broader social impact. This presents a contradiction between the metrics of success of research and its ultimate extrinsic value [1], serving to expose a gulf between ‘strictly objective’ and ‘citizen’ scientists and social scientists [2]; the former believing that research should be objective and independent of external societal influences and the latter whose starting point is that science should benefit society. In recent years the need to link research within broader knowledge utilisation processes has been recognised, or at least accepted, by research councils and increasing numbers of researchers. While some forms of academic enquiry that pushes disciplinary boundaries or that represents ‘blue skies’ thinking remains important, despite being only distally linked to knowledge utilisation, there is little doubt as to the capacity of many other forms of ‘research’ to influence and transform policy and practice (see [3, 4]). In many ways, both systematic reviews and logic models are both borne of such a need for greater knowledge transference and influence. Policy and practice-relevance is integral to most systematic reviews, with the systematic and transparent synthesis of evidence serving to enhance the accessibility of research findings to other researchers and wider audiences [5, 6]. Through an explicit, rigorous and accountable process of discovery, description, quality assessment, and synthesis of the literature according to defined criteria, systematic reviews can help to make research accessible to policy-makers and other stakeholders who may not otherwise engage with voluminous tomes of evidence. Similarly, one of the motivations in evaluation research and programme management for setting out programme theory through a logic model or theory of change was to develop a shared understanding of the processes and underlying mechanisms by which interventions were likely to ‘work’. In the case of logic models this is undertaken through pictorially depicting the chain of components representing processes and conditions between the initial inputs of an intervention and the outcomes; a similar approach also underlies theories of change, albeit with a greater emphasis on articulating the specific hypotheses of how different parts of the chain result in progression to the next stage. This shared understanding was intended to develop across practitioner and program implementers, who may otherwise have very different roles in an intervention, as well as among a broader set of stakeholders, including funders and policy-makers.

As others before us have speculated, there is room for the tools of programme theory and the approach of systematic reviewing to converge, or more precisely, for logic models to become a tool to be employed as part of undertaking a systematic review [7–9]. This is not in dispute in this paper. However, even among audiences engaged in systematic research methods, we remain far from a shared understanding about the purpose and potential uses of a logic model, and even its definition. This has also left us without any protocol around how a logic model should be constructed to enhance a systematic review. In this paper we offer:

1. an account of the way in which logic models are used in the systematic review literature
2. an example of a logic model we have constructed to guide our own work and the documented steps taken to construct this
3. a set of principles for good practice in preparing a logic model

Here, we begin with an outline of the introduction of logic models into systematic reviews and their utility as part of the process.

## The Use of Programme Theory in Review Literature

As understood in the program evaluation literature, logic models are one way of representing the underlying processes by which an intervention effects a change on individuals, communities or organisations. Logic models themselves have become an established part of evaluation methodology since the late 60s [10], although documentation that outlines the underlying assumptions addressing the ‘why’ and ‘for whom’ questions that define interventions is found in literature that dates back further, to the late 50s [11].

Despite being established in evaluation research, programme theory and the use of logic models remains an approach that is underutilised by many practitioners who design, run, and evaluate interventions in the UK [12, 13]. Furthermore, there is a substantial degree of fragmentation regarding the programme theory approach used. Numerous overlapping approaches have been developed within evaluation literature, including ‘logic model’, ‘theory of change’, ‘theory of action’, ‘outcomes chain’, ‘programme theory’ and ‘program logic’ [11, 13]. This very lack of consistency and agreement as to the appropriate tools to be used to conceptualise programme theory has been identified as one reason why, in a survey of 1,000 UK charities running interventions, four-fifths did not report using any formal program theory tool to understand the way in which their interventions effected change on their beneficiaries [12].

Conversely, within systematic reviewing so far, there has been some degree of consensus on the terminology used to represent the processes that link interventions and their outcomes (for example [7, 8, 9]). Many systematic reviews of health interventions tend to settle on a logic model as being the instrument of choice for guiding the review. Alternatively, reviews of international development interventions often include a theory of change, perhaps reflective of the added complexity of such interventions which often take place on a community, policy or systems basis. Logic models and theories of change sit on the same continuum, although a somewhat ‘fuzzy’ but important distinction exists. While a logic model may set out the chain of activities needed or are expected to lead to a chain of outcomes, a theory of change will provide a fuller account of the causal processes, indicators, and hypothesised mechanisms linking activities to outcomes. However, *how* reviews utilise programme theory is relatively unexplored.

### Methods and criteria

To examine the use of logic models and theories of change in the systematic review literature, we examined indicative evidence from two sources. The first of these sources, the Cochrane database publishes reviews that have a largely standardised format that follow guidelines set out in the Cochrane Handbook (of systematic reviews of interventions) [14]. Currently, the handbook itself does not include a section on the use of programme theory in reviews. Other guidance set out by individual Cochrane review groups (of which there are 53, each focussed on a specific health condition or area) may highlight the utility of using programme theory in the review process. For example the Public Health Review Group, in their guidance on preparing a review protocol, describe the way in which logic models can be used to describe how interventions work and to justify a focus of a review on a particular part of the intervention or outcome [15]. Meanwhile, in the 2012 annual Cochrane methods-focussed report, the use logic models was viewed as holding potential to ‘confer benefits for review authors and their readers’ [16] and logic models have also been included in Cochrane Colloquium programme [17]. However, a definitive recommendation for use is not found, at the time of writing, in standard guidance provided to review authors. The second source, the 3ie database includes reviews with a focus on the effectiveness of social and economic interventions in low- and middle- income countries. The database includes reviews that have been funded by 3ie as well as those that are not directly funded but that nevertheless fall within its scope and are deemed to be of sufficient rigour. While the use of programme theory does not form part of the inclusion criteria, its use is encouraged in good practice set out by 3ie [18] and a high degree of importance is attributed to its use in 3ie’s criteria for awarding funding for reviews [19].

To obtain a sample of publications, we searched the Cochrane Library for systematic reviews and protocols and for material that included either the phrase ‘logic model’ or ‘theory of change’ occurring anywhere, published between September 2013 and September 2014 (over this period a total of 1,473 documents were published in the Cochrane Library). We also searched the 3ie (International Initiative for Impact Evaluation) database of systematic reviews published in 2013, and manually searched publications for the phrases ‘logic model’ or ‘theory of change’. Both searches were intended to provide a snapshot of review activity through capturing systematic review publications occurring over the course of a year. For the 3ie database, it was not possible to search by month, therefore we searched for publications by calendar year; to ensure that we obtained a full sample for a year we selected 2013 as our focus. For the Cochrane database, in order to obtain a more recent snapshot of publications to reflect current trends, we opted to search for publications occurring over a year (13 months in this case). All reviews and protocols of reviews that fell within the search parameters were analysed.

In the Cochrane database, over this period, four reviews and ten protocols were published that included the phrase ‘logic model’; while two protocols were published that included the phrase ‘theory of change’. It should be noted therefore that, certainly within reviews of health topics that adhere to Cochrane standards, that neither tool has made a substantial impact in this set of literature. This is likely to reflect the mainly clinical nature of many Cochrane reviews–among the 8 publications that were published through the public health group (all of which were protocols), 5 included mention of programme theory. Within the 3ie database of international development interventions, 53 reviews and protocols were published in 2013 (correct as of December 2014), of which 24 included a mention of either a logic model or a theory of change.

We developed a template for summarising the way in which logic models were used in the included protocols and systematic reviews based on the different stages of undertaking systematic reviews [6] and the potential usage of systematic reviews as identified by Anderson and colleagues and Waddington and colleagues [7, 18], who in the former case describe logic models as being tools that can help to (i) scope the review; (ii) define and conduct the review; and (iii) make the review relevant to policy and practice including in communicating results. These template constructs also reflected the way in which logic model usage was described in the publications, which was primarily shaped by reporting conventions for protocols and reports published in Cochrane and 3ie (although the format for the latter source is less structured). Criteria around the constructs included in the template were then defined before two reviewers (see S1 Table. Data Coding Template) then independently assessed the use of logic model within the reviews and protocols published; the reviewers then met to discuss their findings. What this template approach cannot capture is the extent to which using a logic model shaped the conceptual thinking of the review teams, which, as discussed later in this paper, is one of the major contributions of using a logic model framework.

While both databases cover two different disciplines, allowing us to make comparisons between these, there may be some who argue that through having rigidly enforced methodological guidelines in the production of reviews, that we are unlikely to encounter innovative approaches to the use of programme theory in the reviews and protocols included here. This is a legitimate concern and is a caveat of the results presented here, although even among these sources we observe considerable diversity in the use of programme theory as we describe in our results.

### Results: how are logic models and theories of change used in systematic reviews?

Looking first at publications from the Cochrane database and the two studies that included some component examining ‘theories of change’, the first of these described ‘theory of change’ in the context of synthesising different underlying theoretical frameworks [20] while the second used ‘theory of change’ in the context of describing a particular modality of theory [21]. Meanwhile, logic models were incorporated in a variety of ways; most frequently, they have been used as a shorthand way to describe how interventions are expected to work, outlining stages from intervention inputs through to expected outcomes at varying levels of detail (Table 1).

**Table 1:** Use of Logic Models in Cochrane Library Publications.

Study	Protocol or Report	How the logic model was used in the review
Chamberlain et al. [22]	Report	Used to describe how the intervention might work; Used to guide sub-group analyses
Glenton et al. [23]	Report	Used to describe how the intervention might work; Developed from existing theories of change/logic models/frameworks; Used to guide sub-group analyses; Logic Model used to structure qualitative synthesis; Used to synthesise qualitative and quantitative evidence; Did or plan to revise at the end of the review; Described as being constructed through consensus building
Langford et al. [24]	Report	Used to describe how the intervention might work; Used to guide type of intervention (selection criteria); Described as being constructed through consensus building
Burns et al. [25]	Protocol	Used to describe how the intervention might work; Used to guide type of intervention (selection criteria)
Costello et al. [26]	Protocol	Used to describe how the intervention might work; Used to guide type of intervention (selection criteria)
Gavine et al. [27]	Protocol	Used to describe how the intervention might work;
Kuehnl et al. [28]	Protocol	Used to describe how the intervention might work;
Land et al. [29]	Protocol	Used to describe how the intervention might work; Developed from existing theories of change/logic models/frameworks;
Langbecker et al. [30]	Protocol	Used to describe how the intervention might work; Described as being constructed through consensus building
Michelozzi et al. [31]	Protocol	Used to describe how the intervention might work
Peña-Rosas et al. [32]	Protocol	Used to describe how the intervention might work; Developed from existing theories of change/logic models/frameworks
Ramke et al. [33]	Protocol	Used to describe how the intervention might work; Developed from existing theories of change/logic models/frameworks; Did or plan to revise at the end of the review;
Sreeramareddy and Sathyanarayana [34]	Protocol	Used to describe how the intervention might work

Search included full reviews and protocols published over thirteen months between September 2013 and September 2014 that included ‘logic model’ in the text.

In around half of reports and protocols, the authors described in some form how they planned to, or did in fact use, their logic model in the review. Nevertheless, in the remainder of publications (all of which were protocols), the logic model was presented as a schematic to describe how the intervention may work and was not explicitly referred to further as a tool to guide the review process. Two Cochrane review protocols explicitly outlined the way in which the logic model would be used as a reference tool when considering the types of intervention that would be within the scope of the review [25, 26]; this was also described in a full review [24]. We identified three publications where it was suggested that the logic model was developed through an iterative process where consensus was developed across the review team [23, 24, 30].

Two Cochrane reviews described how the logic model was used to determine the subgroup analyses a priori [22, 23], helping to avoid some of the statistical pitfalls of running post-hoc sub-group analyses [35]. For example in their review of psychosocial smoking cessation interventions for pregnant women, Chamberlain and colleagues [22] developed their logic model from a synthesis of information collected before the review process began, with the express purpose of guiding analyses and stating sub-group analyses of interest. In Glenton’s study, a revision to the logic model they originally specified, based on the review findings was also viewed as being a useful tool to guide the sub-group analyses of future reviews. However, none of the protocols (as opposed to reviews) from the Cochrane database explicitly mentioned that the logic model had been used to consider which sub-group analyses should be undertaken. The review and protocol by Glenton et al. [23] and Ramke et al. [33] respectively provided the only examples where the logic model was to be revised iteratively during the course of the review based on review findings. Of the three Cochrane reviews included in Table 1, Glenton and colleagues’ study [23] can be considered the one to have used a logic model most comprehensively as a dynamic tool to be refined and used to actively the synthesis of results in the review. The authors describe a novel use of the logic model in their mixed methods review as being a tool to describe mechanisms by which the intervention barriers and facilitators identified in the qualitative synthesis could impact on the outcomes assessed quantitatively in their review of programme effectiveness.

Among the studies extracted from the 3ie database, the terminology was weighted towards the ‘theories of change’ as opposed to ‘logic models’ (as expected, based on the guidance provided). Out of the 24 studies that were included (Table 2), fourteen included a Logic Model and nine included a Theory of Change, while one report used both terms. Despite more studies including mention or an actual depiction of a theory of change or logic model, this body of literature shared the same limitations around the extent of use of programme theory as a tool integral to the review process. The majority of studies used a Theory of Change/Logic Model to describe their overall conceptual model or how they viewed the intervention or policy change under review would work, although this was reported at different stages of the review. Of the eleven protocols that were included, eight explicitly mentioned that they planned to return to their model at the end of the review, emphasising the use of programme theory tools as tools to help design the review *and* communicate the findings in this field. For example, in Willey and colleagues’ review of approaches to strengthen health services in developing countries [59], the Logic Model was updated at the end of the review to reflect the strength of the evidence discovered for each of the hypothesised pathways. Seven of the twenty protocols and studies described how a theory of change/logic model would be used to guide the review in terms of search strategy or more generally as a reference throughout the screening and other stages. Finally, two publications [48, 52] described how they would use a theory of change as the basis for synthesising qualitative findings and two described how they would use a logic model/theory of change to structure sub-group meta analyses in quantitative syntheses [48, 58]; both of these two latter protocols described how programme theory would be used at a number of key decision points in the review itself.

**Table 2:** Use of Logic Models and Theories of Change in reviews of international development published on 3ie.

Study	Protocol or Report	Logic Model or Theory of Change	How the logic model was used in the review
Baird et al. [36]	Report	TOC	Used to describe how the intervention might work
Brody et al. [37]	Protocol	TOC	Used to describe how the intervention might work; Did or plan to revise at the end of the review
Cirera et al. [38]	Report	Both	Used to describe how the intervention might work; Used to guide type of intervention (selection criteria); Did or plan to revise at the end of the review
Coren et al. [39]*	Report	LM	Used to describe how the intervention might work; Did or plan to revise at the end of the review
Giedon et al. [40]	Report	TOC	Used to describe how the intervention might work (post-hoc)
Gonzalez et al. [41]	Protocol	TOC	Used to describe how the intervention might work; Did or plan to revise at the end of the review
Higginson et al. [42]	Protocol	LM	Used to describe how the intervention might work; Developed from existing theories of change/logic models/frameworks; Did or plan to revise at the end of the review
Higginson et al. [43]	Protocol	LM	Used to describe how the intervention might work; Did or plan to revise at the end of the review
Kingdon et al. [44]	Report	TOC	Used to describe how the intervention might work
Kluve et al. [45]	Protocol	TOC	Used to describe how the intervention might work (post-hoc)
Loevinsohn et al. [46]	Report	TOC	Used to describe how the intervention might work
Lynch et al. [47]	Report	LM	Used to describe how the intervention might work; Used to guide type of intervention (selection criteria)
Molina et al. [48]	Protocol	TOC	Used to describe how the intervention might work; Used to guide type of intervention (selection criteria);
Posthumus et al. [49]*	Report	TOC	
Samarajiva et al. [50]	Protocol	TOC	Used to describe how the intervention might work; Used to guide type of intervention (selection criteria); Extraction of study characteristics; Did or plan to revise at the end of the review
Samii et al. [51]	Protocol	TOC	Used to describe how the intervention might work; Did or plan to revise at the end of the review
Seguin et al. [52]	Report	TOC	Used to describe how the intervention might work; Used to synthesise qualitative and quantitative evidence; Did or plan to revise at the end of the review
Spangaro et al. [53]*	Report	TOC	
Stewart et al. [54]	Protocol	TOC	Used to describe how the intervention might work (post-hoc)
Tripney et al. [55]	Report	LM	Used to describe how the intervention might work
Tripney et al. [56]	Protocol	LM	Used to describe how the intervention might work; Did or plan to revise at the end of the review
Turley et al. [57]	Report	LM	Used to describe how the intervention might work
Welch et al. [58]	Protocol	LM	Used to describe how the intervention might work; Used to guide type of intervention (selection criteria); Used to guide sub-group analyses; Used to structure qualitative synthesis; Did or plan to revise at the end of the review
Willey et al. [59]	Report	LM	Used to describe how the intervention might work; Did or plan to revise at the end of the review

Search included full reviews and protocols published in 2013; only those that included a mention of ‘theory of change’ or ‘logic model’ are included here.

TOC = Theory of Change; LM = Logic Model.

*Did not include an actual depiction.

Among the Cochrane and 3ie publications, few reviews or protocols described the logic model as being useful in the review initiation, description of the study characteristics or in assessing the quality and relevance of publications. Three Cochrane protocols and one Cochrane review described using existing logic models in full or examining components of existing logic models or reviews to develop their own while one in our sample of international development systematic reviews did so. Most authors appear to develop their own logic models afresh, and largely in the absence of guidance around good practice around the use of logic models. As Glenton and colleagues describe there is “no uniform template for developing logic models, although the most common approach involves identifying a logical flow that starts with specific planned inputs and activities and ends with specific outcomes or impacts, often with short-term or intermediate outcomes along the way” ([23]; p13).

## Developing a Logic Model: A Worked Example from School Based Asthma Interventions

**Fig 1:**
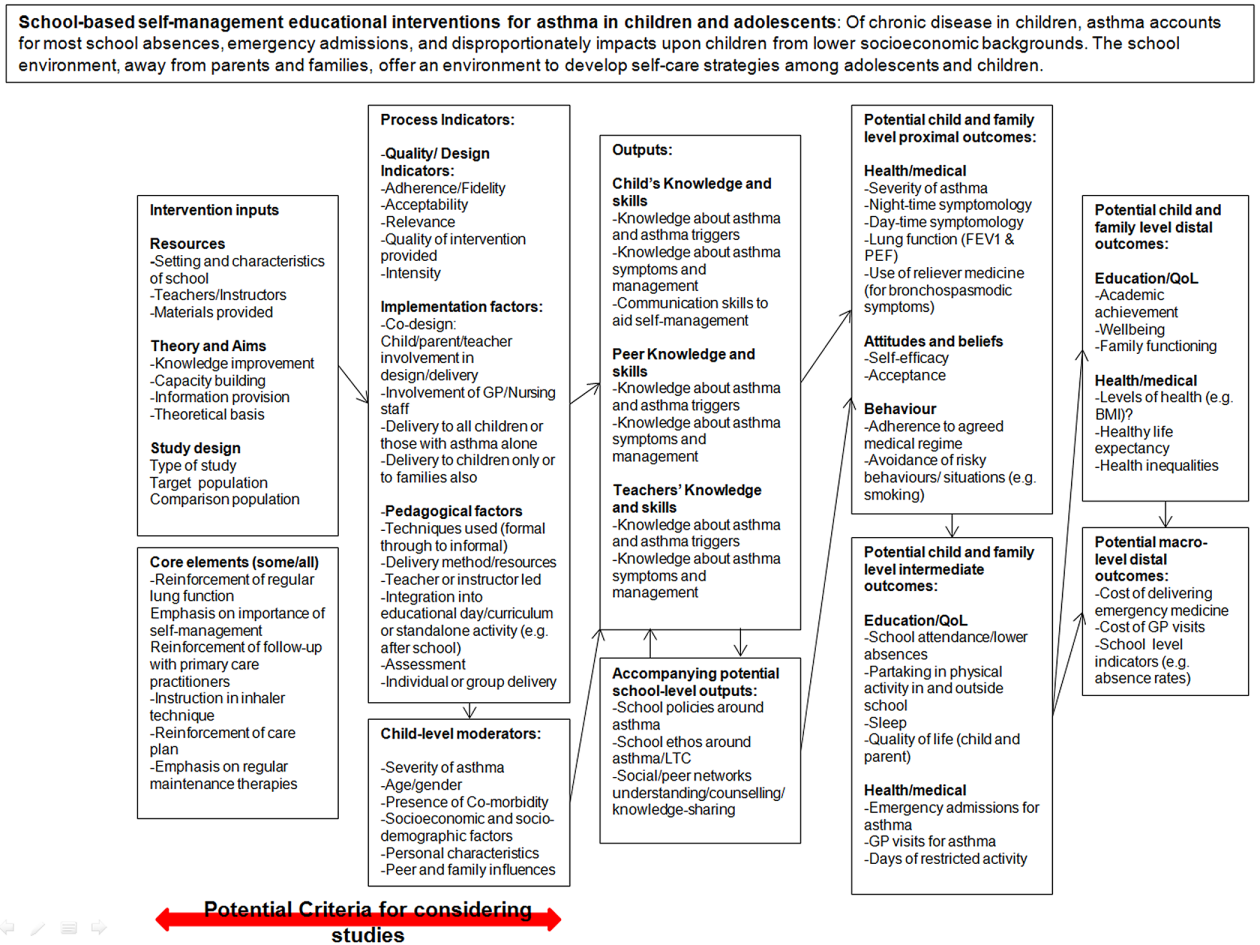
First iteration of Logic Model (developed by one review team member).

**Fig 2:**
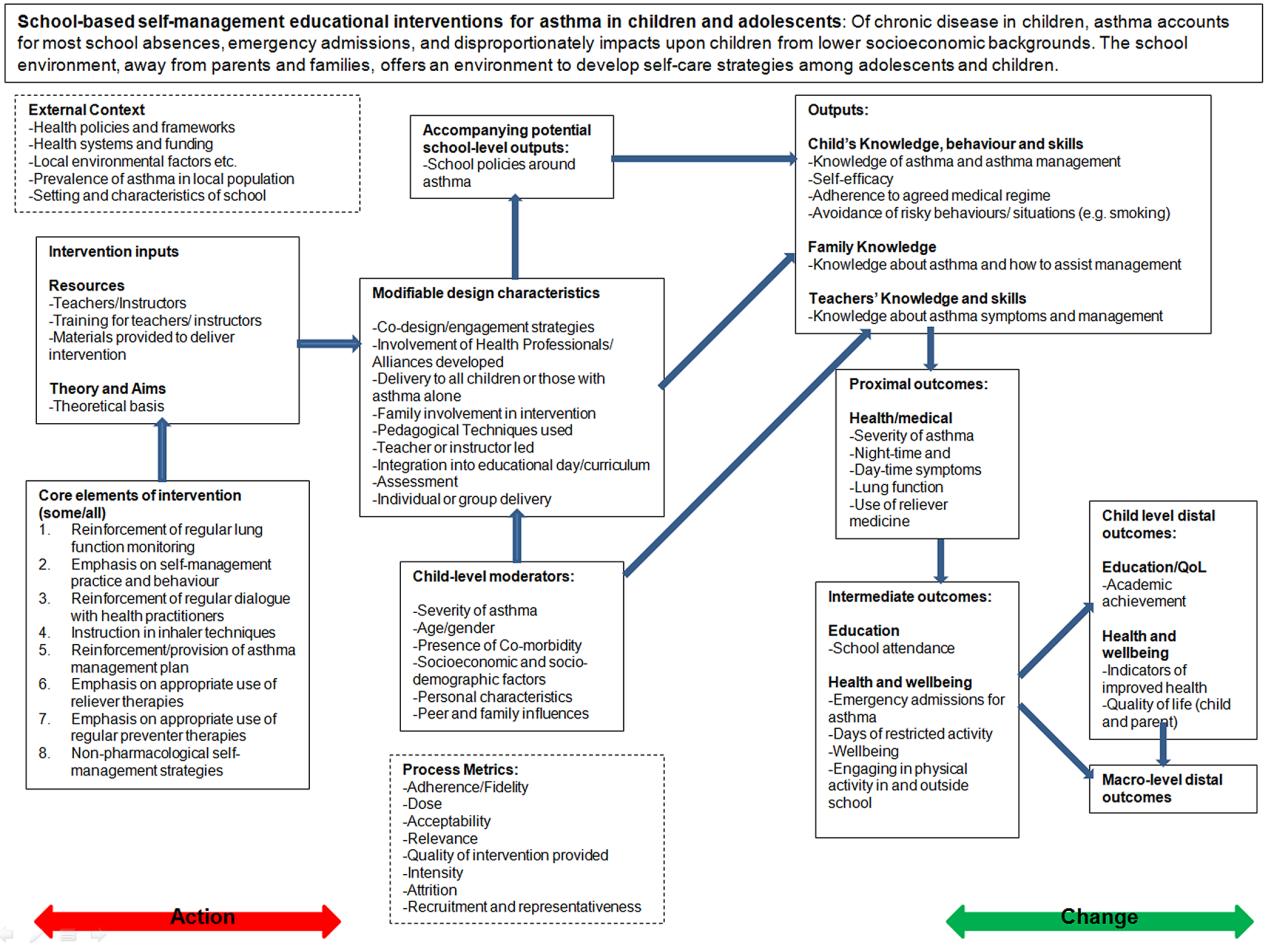
Second iteration of Logic Model (developed by two review team members).

**Fig 3:**
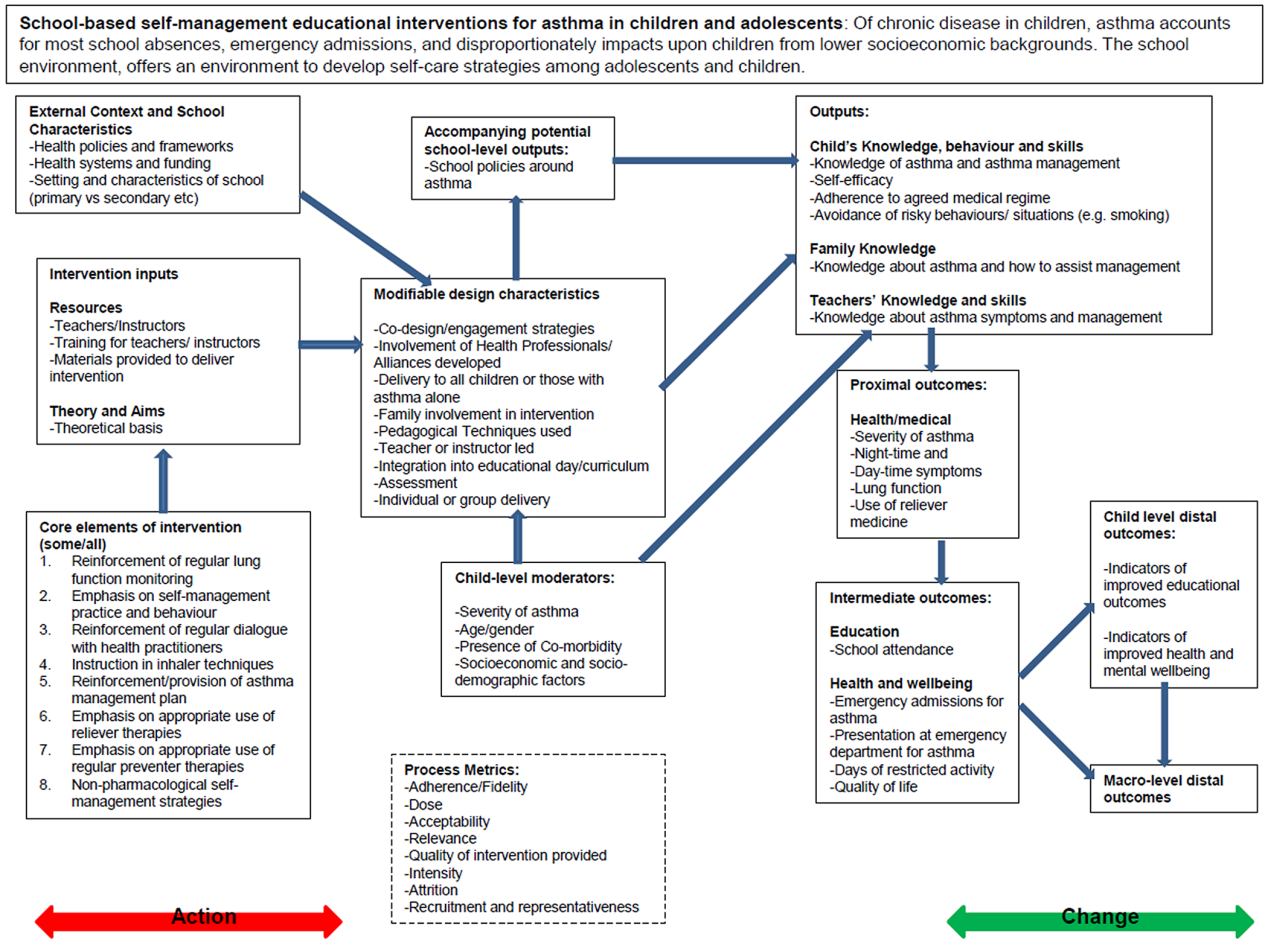
Third iteration of Logic Model (agreed by review team).

The second aim of this paper is to provide an example of the development of a logic model in practice. The logic model we describe is one developed as part of a systematic review examining the impact of school based interventions focussing on the self-management of asthma among children. This review is being carried out by a multidisciplinary team comprising team members with experience of systematic reviewing as well as team members who are trialists with direct experience in the field of asthma and asthma management. Of particular interest in this review are the modifiable intervention factors that can be identified as being associated with improvements in asthma management and health outcomes. The evidence will be used directly in the design of an intervention that will be trialled among London school children. Our approach was to view the development of the logic model as an iterative process, and we present three different iterations (Figs 1–3) that we undertook to arrive at the model we included in our review protocol [60]. Our first model was based on pathways identified by one reviewer through a summary examination of the literature and existing reviews. This was then challenged and refined through the input of a second reviewer and an information scientist, to form a second iteration of the model. Finally, a third iteration was constructed through the input of the wider review team, which included both methodological specialists and clinicians. These steps are summarised in Box 1 and are described in greater detail in the sections below. The example provided here is one that best reflects a process driven logic model where the focus is on establishing the key stages of interest and using the identified processes to guide later stages of the review. An alternative approach to developing a logic model may be to focus more on the representation of systems and theory [61]; although this approach may be better placed to support reviews of highly complex interventions (such as many of the international development reviews described earlier) or reviews that are more methodological than empirical in nature.

**Box 1. Summary of steps taken in developing the logic model for school based asthma interventions.**

1. Synthesis of existing logic models in the field
2. Reviewer 1 identified distal outcomes
3. Working backwards, reviewer 1 then identified the necessary preconditions to reach distal outcomes; from distal outcomes intermediate and proximal level outcomes were then identified
4. Once outcomes had been identified, the outputs were defined (necessary pre-conditions but not necessarily goals in themselves); on completion the change part of the model was complete in draft form
5. Modifiable processes were then specified these were components that were not expected to be present in each intervention included in the review
6. Continuing to work backwards, intervention inputs (including core pedagogical inputs) were then specified. These were inputs that were expected to be present in each intervention included in the review, although their characteristics would differ between studies
7. In addition, external factors were identified as were potential moderators
8. Reviewer 1 and 2 then worked together to redevelop the model paying particular attention to clarity, the conceptual soundness of groupings and the sequencing of aspects
9. The review team and external members were asked to comment on the second iteration, and later agree a revised version 3. This version would provide the structure for some aspects of quantitative analyses and highlight where qualitative analyses were expected to illuminate areas of ambiguity.
10. The final version was included in the protocol with details on how it would be used in later stages of the review, including the way in which it would be transformed, based on the results uncovered, into a theory of change.
11. Consider undertaking additional/supplementary steps12.

### Step 1, examination and synthesis of existing logic models

The first step we took in developing our logic model was to familiarise ourselves with the existing literature around the way in which improved self-management of asthma leads to improved outcomes among children and the way in which school-based learning can help to foster these. Previous systematic reviews around this intervention did not include a logic model or develop a theory of change but did help to identify some of the outcomes of self-management educational interventions. These included improved lung, self-efficacy, absenteeism from school, days of restricted activity, and number of visits to an emergency department, among others (see [62]). A logic model framework helped to order these sequentially and separate process outputs from proximal, intermediate and distal outcomes. Other studies also pointed towards the school being a good site for teaching asthma self-management techniques among children for several reasons, including the familiar environment for learning that it provides for children, and the potential for identification of large numbers of children with asthma at a single location [63–65]. Some individual studies and government action plans also included logic models showing how increased education aimed at improving self-management skills was expected to lead to improvements in asthma outcomes (for example [66, 67, 68]). This evidence was synthesised and was found to be particularly useful in helping to identify some of the intervention processes taking place that could lead to better asthma outcomes, although these were of varying relevance to our specific situation of interest in terms of school-based asthma interventions, as well as being very heavily shaped by local context. We adopted an aggregative approach to the synthesis of the evidence at this point, including all information which was transferable across contexts [69]. After examining the available literature, the first reviewer was able to proceed with constructing a first draft of the logic model.

### Step 2, identification of distal outcomes

Reviewer 1 started with the identification of the very distal outcomes that could change as a result of school-based interventions aimed at improving asthma self-management. From these outcomes the reviewer worked backwards and identified the necessary pre-conditions to achieving these to develop a causal chain. Identifying this set of distal outcomes was analogous to questioning why a potential funding, delivery or hosting organisation (such as a school or health authority) may want to fund such an intervention–the underlying goals of running the intervention. In this case, these outcomes could include potential improvements in population-level health, reductions in health budgets and/or potential increases in measures of school performance (Fig 1). After identifying these macro-level outcomes, we identified the distal child level outcomes which were restricted to changes in children’s outcomes that would only be perceptible at long-term follow-up. These included changes in quality of life and academic achievement, which we identified as being modifiable only after sustained periods of behaviour change and a reduction in the physical symptoms of asthma.

### Step 3, identification of intermediate and proximal outcomes

Next, the reviewer 1 outlined some of the intermediate outcomes, those changes necessary to achieve the distal outcomes. Here our intermediate changes in health were based on observations of events, including emergency admissions and limitations in children’s activity over a period of time (which we left unspecified). The only intermediate educational outcome was school attendance, and we identified this as being the only (or at least main) pathway through which we may observe (distal) changes in academic achievement as a result of school-based asthma interventions. Working backwards, our proximal outcomes were defined those pre-conditions necessary to achieve our intermediate outcomes; these revolved around health symptoms and behaviour around asthma and asthma management. We expect these to be observable shortly after the intervention ends (although may be measured at long-term follow-up). The intention is for the systematic review to be published as a Cochrane review which requires the identification of 2–3 primary outcomes and approximately 7 outcomes in total, which in our case helped to rationalise the number of outcomes we included, which left unbounded could have included many more.

### Step 4, identification of outputs

Finally in the ‘change’ section of the logic model (see Fig 2), we then specified the outputs of the intervention, which we define here as those aspects of behaviour or knowledge that are the direct focus for modification within the activities of the intervention, but are unlikely to represent the original motivations underlying the intervention. Our outputs are those elements of the intervention where changes will be detectable during the course of the intervention itself. Here increased knowledge of asthma may be a pre-condition for improved symptomology and would have a direct focus within intervention activities (outputs), but increased knowledge in itself was not viewed as an likely underlying motivation of running the intervention. A different review question may prioritise improved knowledge of a health condition, and view increased knowledge as an end-point in itself.

### Step 5, specification of modifiable intervention processes

To aid in later stages of the review we placed the modifiable design characteristics in sequence after intervention inputs, as we view these as variants that can occur once the inputs of the intervention have been secured. Separating these from standard intervention inputs was a useful stage when it came to considering the types of process data we might extract and in designing data extraction forms. The number of modifiable design characteristics of the intervention specified was enhanced by examining some of the literature described earlier as well as through discussions with members of the review team who were most involved with designing the intervention that will take place after the review.

### Step 6, specification of intervention inputs

Standard intervention inputs were specified as were the ‘core elements of the intervention’. These core elements represent the pedagogical focus of the intervention and form some of the selection criteria for studies that will be included, although studies will differ in terms of the number of core elements that are included as well as the way in which these are delivered. Studies that do not include any of these core elements were not considered as interventions that focus on the improvement asthma self-management skills.

### Step 7, specification of intervention moderators including setting and population group

Finally, child level moderators (population characteristics) and the characteristics of the schools receiving the intervention were specified (context/setting characteristics). Specifying these early-on in the logic model helped to identify early-on the type of subgroup analyses we would conduct to investigate any potential sources of heterogeneity.

### Step 8, share initial logic model, review and redraft

Reviewer 1 shared the draft logic model with a second member of the team. Of particular concern in this step was to establish consensus around the clarity, conceptual soundness of the groupings, the sequencing of the change part of the model, and the balance between meeting the design needs of the intervention and the generalisability of the findings to other settings. With respect to the latter, the second reviewer commented that specifying reductions in health budgets reflected our own experiences of the UK context, and may not be appropriate for all healthcare contexts likely to be included in our review. Therefore, Fig 2 in our second iteration only acknowledges that macro-level (beneficial) changes can be observed from observing changes in the distal outcomes of children, but we do not specify what these might be. At this stage it was helpful to have the first reviewer working alongside a second member of the review team who had greater expertise and knowledge of the design and delivery health-based interventions and who was working directly alongside schools in the preliminary stages of data collection around the intervention itself. Figs 1–3 show the development of the logic model across iterations. This second iteration had a clearer distinction between the action and change aspects of the logic model, and had refined the number of outcomes that would be explicitly outlined, which had implications for the search strategy. The action part of the model was also altered to better differentiate parts of the model that represented implementation processes from parts of the model that represented implementation measures or metrics.

### Step 9, share revised logic model with wider group, review and redraft

The draft logic model was shared among the wider review team and to an information scientist with comments sought, particularly around those aspects of step 8 that had been the source of further discussion. The review team were asked specifically to input on the content of the different sections of the logic model, the sequencing of different parts, and the balance between meeting the design needs of the intervention and the generalisability of the findings to other settings. Input was sought from an information scientist external to review to ensure that the model adequately communicated and captured the body of literature that the review team aimed to include, and helped to make certain that the model was interpretable to those who were not directly part of the review team. For the third (and final) iteration, views were also sought about whether the main moderating factors across which the team might investigate sources of heterogeneity in meta analyses were included, or for those that would be identified through earlier qualitative analyses, that these were adequately represented. Once these views were collated, the third iteration was produced and agreed. The third iteration better represents the uncertainty in terms of processes that may be uncovered during qualitative analyses and the way in which these will be used to investigate heterogeneity in subgroup analyses in the quantitative (meta) analysis.

### Step 10, present the final logic model in the protocol

The final version was included in the protocol with details on how it would be used in later stages of the review. At the end of the review, we intend to return to the logic model and represent those factors that are associated with successful interventions from the quantitative and qualitative analyses in a theory of change.

### Potential additional and supplementary steps that could be taken elsewhere

*Greater consultation or active collaboration with additional stakeholders* on the logic model may be beneficial, particularly for complex reviews involving system-based interventions where different stakeholders will bring different types of knowledge [8, 70]. There may also be merit in this approach at the outset in situations where the review findings are intended to inform on an intervention in a known setting, and to ensure that the elements that will enhance the applicability or transferability of the intervention are represented. In the example given here, as there were members of the review team who were both taking part in the review and the design of the intervention, there was less of a need to undertake this additional stage of consultation, and elements such as the presence or change in school asthma policies were included in the logic model to reflect the interests of the intervention team.

*Produce further iterations of the logic model*: When there is less consensus among the review team than was the case here, when there are greater numbers of stakeholders being consulted, or when the intervention itself is a more complex systems-based intervention; there may be a need to produce further multiple iterations of the logic model. In programme evaluation, logic models are considered to be iterative tools that reflect cumulative knowledge accrued during the course of running an intervention [11]. While the exact same principle doesn’t apply in the case of systematic reviews, a greater number of iterations may be necessary in order to produce a logic model to guide the review, for example to reflect the different forms of knowledge different stakeholders may bring. Where there are parts of the logic model that are unclear at the outset of a review, or in situations where there is an insurmountable lack of consensus and only the review findings can help to clarify the issue, these can be represented in a less concrete way in the logic model, for example the processes to be examined in our own review in Fig 3.

*Multiple logic models*: There may also be a need to construct multiple logic models for large interventions to reflect the complexity of the intervention, although it may also be the case that such a large or complex question may be unsuitable for a single review but would instead fall across multiple linked reviews. However, where the same question is being examined using different types of evidence (mixed method review), multiple logic models representing the same processes in different ways could be useful–for example a logic model focussing on theory and mechanistic explanations for processes in addition to a logic model focussing on empirical expected changes may be necessary for certain forms of mixed methods reviews (dependent on the research question). In other cases, the review may focus on a particular intervention or behaviour change mechanism within a (small) number of defined situations–for example a review may focus on the impact of mass media to tackle public health issues using smoking cessation, alcohol consumption and sexual health as examples. The review question may be focussed on the transferability of processes between these public health issues but in order to guide the review itself it may be necessary to produce a separate logic model for each public health issue, which could be synthesised into a unified theory of change for mass media as an intervention at a later stage.

#### Using the logic model in the review

The logic model described in this paper is being used to guide a review that is currently in progress and as such we are not able to give a full outline of its potential use. Others in the literature before us have described logic models as having added value to systematic reviewers when (i) scoping the review (refining the question; deciding on lumping or splitting a review topic; identifying review components); (ii) defining and conducting the review (identifying the review study criteria; guiding the search strategy; rationale behind surrogate outcomes; justifying sub-group analyses); (iii) making the review relevant to policy and practice (structuring the reporting of results; illustrating how harms and feasibility or connected with interventions, interpreting the results based on intervention theory) [7, p35]. Others still have emphasised the utility of a logic model framework in helping reviewers to think conceptually through illustrating the influential relationships and components from inputs to outcomes, suggesting that logic models can help reviewers identify testable hypotheses to focus upon [8, 71]; they have also speculated that a logic models could help to identify the parameters of a review as an addition to the well-established PICO framework [8, 9].

Our own experience of using the logic model (Fig 3) in a current systematic review to date is summarised in Table 3 below; which focuses on additions to the uses suggested elsewhere. While the additional description below provides an indication as to the potential added value of using a logic model, the use of a logic model has not be without its challenges. Firstly, the use of logic models is relatively novel within the systematic review literature (and even in program theory literature, as discussed earlier), and initially there was some apathy towards the logic model, even within the review team. Secondly, while we agree that a logic model could be used to depict the PICO criteria [8, 9], our own logic model did not include a representation of ‘C’, the comparator, as this was the usual care provided across different settings, which could vary substantially. Others may also experience difficulties in representing the comparison element in their logic models. Finally, all of the utilities of the logic model described here and elsewhere are not unique qualities or contingent to using a logic model, but using a logic model accelerates these processes and brings about a shared understanding more quickly; for example development of exclusion criteria is not contingent on having a logic model, but rather that the logic model facilitates the process of identifying inclusion and exclusion criteria more rationally, and helps depict some of the reasoning underlying review decisions. Practically, where the logic model has its advantages is in aiding the initial conceptual thinking around the scope of interventions, its utility in aiding decisions about individual parts of the intervention within the context of the intervention as whole, its flexibility and its use as a reference tool in decision-making, and in communication across the review team.

**Table 3:** Use of logic model for school-based asthma interventions

Systematic review stage (see [6])	Added value	Processes undertaken/detail
**Review initiation**	Thinking conceptually	The logic model provided a useful tool when considering whether the relationships we were specifying were suitable for review in a single mixed-method review i.e. whether the scope of the review was conceptually sound and feasible for a single review.
	Gain initial understanding of the way in which the intervention is likely to work	Through the examination of existing logic models we were able to gain an initial understanding of the way in which the intervention was likely to work and the sequencing of likely inputs, processes, outputs and outcomes.
**Review question and methodology**	Defining the scope of the review	The logic model was a useful tool in enabling the review team to think conceptually around the processes that might be undertaken within a review and helped us to clarify the characteristics of studies which may reasonably be considered to be process evaluations. This helped distinguish between a process evaluation and a qualitative views study focussed on outcomes.
	Identifying points of uncertainty	The logic model helped the review team identify parts of the review where the evidence was uncertain or where further direction would emerge during the course of undertaking the review.
	Addressing theory	Developing the logic model was also a useful exercise in reinforcing that we were not relying on any particular theory a priori, but instead we were synthesising evidence that could provide support for different forms of mechanistic explanations at a later stage, which we would be able to communicate through a theory of change.
**Search strategy**	Developing exclusion criteria	The logic model framework allowed us to identify the intervention components and separate these into ‘core’ elements which may form part of the inclusion/exclusion criteria, some of expected elements/inputs of the intervention which did not constitute core inclusion/exclusion criteria (for instance training for instructors), and some of the additional non-standard modifiable processes which might be of interest but were not core inclusion/exclusion criteria in of themselves and were to be extracted from the included studies.
	Informing the search strategy	The logic model helped the review team to identify the breadth of different databases from which studies needed to be identified by discipline through examining the types of relationships that were being specified in the model. For example, the development of self-efficacy as an intermediary step in the model helped to reinforce that the review needed to include literature from psychology and that this needed to be reflected in the selection of databases.
**Description of study characteristics**	Developing data extraction forms	The logic model informed the first drafts of the data extraction forms being used on the review, and was particularly useful when it came to designing the data extraction form for process evaluations when it became apparent that data extraction needed to reflect both those processes that were certain we wanted to capture information on as well as those processes we were not able to pre-specify.
**Project management and review administration**	Communication tool across review team	We are currently in the process of screening the results but the logic model has been used as a quick communication tool in helping to settle disagreements between reviewers in deciding whether to include or exclude studies. It has also been used as a communication tool within the team helping to justify decisions around the scope of the literature included.
	Project Management/ Sequencing the review	The logic model was also useful in helping to communicate points of uncertainty and where ‘dependencies’ in the review lay—in other words those points of uncertainty in the review which needed to be resolved before later stages could proceed. From a project management perspective this helped to identify the sequencing necessary for different parts of the review.

## Developing Elements of Good Practice for the Use of Logic Models and Theories of Change

The earlier analysis suggests that many systematic review authors tend to use programme theory tools to depict the conceptual framework pictorially, but may not view either a logic model or theory of change as integral review tools. To prevent logic models and theories of change being included in reviews and protocols as simply part of a tick-box exercise, there is a need to develop good practice on how to use programme theory in systematic reviews, as well as developing good practice on how to develop a logic model/theory of change. This is not to over-complicate the process or to introduce rigidity where rigidity is unwelcome, but to maximise the contribution that programme theory can make to a review.

Here we introduce some elements of good practice that can be applied when considering the use of logic models. These are derived from (i) the literature around the use of logic models in systematic reviews [7, 8, 17]; (ii) the broader literature around the use of theory in systematic reviews [72, 73]; (iii) our analyses contrasting the suggested uses of logic models in systematic reviews with their actual use (section1); (iv) the use of logic models in program theory literature [11, 13]; as well as broader conceptual debates in systematic review and program theory literature. These principles draw from the work of Anderson and colleagues and Baxter and colleagues as well as our own experiences, but are unlikely to represent an exhaustive list as there is a need to maintain a degree of flexibility in the development and use of logic models. Our main concern is that logic models in the review literature appear to be used in such a limited way that a loose set of principles, such as those proposed here, can be applied with little cost in terms of imposing rigidity but with substantial impact in terms of enhanced transparency in use and benefit to the review concept, structure and communication.

### A logic model is a tool and as such its use needs to be described

Logic models provide a framework for ‘thinking’ conceptually before, during and at the end of the review. In addition to the uses highlighted earlier by Anderson and Waddington [7, 18], our own experiences of using the logic model on our review has emphasised the utility of a logic model in: (i) clarifying the scope of the review and assessing whether a question was too broad to be addressed in a single review; (ii) identifying points of uncertainty that could become focal points of investigation within the review; (iii) clarification of the scope of the study and particularly in distinguishing between different forms of intervention study design (in our own case between a process evaluation and a qualitative outcomes evaluation); (iv) ensuring that there is theoretical inclusivity at an early stage of the review; (v) clarifying inclusion and exclusion criteria, particularly with regards to core elements of the intervention; (vi) informing the search strategy with regards to the databases and scholarly disciplines upon which the review may draw literature; (vii) a communication tool and reference point when making decisions about the review design; (viii) as a project management tool in helping to identify dependencies within the review. Sharing the logic model with an information scientist was also a means of communicating the goals of the review itself while examination of existing logic models was found to be a way of developing expertise around how an intervention was expected to work. Use of a logic model has also been linked with a reduced risk of type III errors occurring, helping to avoid conflation between errors in the implementation and flaws in the intervention [17, 74].

Summarising our own learning around the uses of the logic model and the uses identified by others (primarily Anderson) for their use as a tool in systematic reviews in Table 4 highlights that a logic model may have utility primarily at the beginning and end of the systematic review, and may be a useful reference tool throughout.

**Table 4:** Potential utility of a logic model in a systematic review by review stage.

Stage (see [6])	Stage processes	Potential utility of a logic model
Review initiation	Forming review team; Engage stakeholders	Possible added value as a tool for early communication with stakeholders
Review question and methodology	Formulate question, conceptual framework & approach	Added value in defining and refining conceptual aspects of the review including: scope, boundaries, study types and methods, appropriateness, PICO criteria, identification of uncertainties and areas of focus, understanding how the review will work
Search strategy	Search and screen for inclusion using eligibility criteria	Developing search strategy including defining concepts, making choices around databases, the number/types of searches, developing eligibility criteria for results, identifying appropriate study designs
Description of study characteristics	Code to match or build a conceptual framework	Aid in the design of data extraction forms
Quality relevance and appraisal	Apply quality appraisal criteria	Unclear
Synthesis	Use conceptual framework, study codes & quality judgements	Identify and justify outcomes and as aid to pre-specifying and justifying sub-group analyses; identify potential areas for focussing on during narrative synthesis or focussing parts of the qualitative synthesis
Using reviews	Interpret & communicate findings with stakeholders	As a dissemination tool when communicating review findings; as a tool for communicating new knowledge and theories deriving from the review in the form of a theory of change
Project Management	Throughout	Identifying dependencies and sequences in undertaking the review; communication tool throughout the review

Our analyses suggest that the use of logic models has faltered and our earlier review of the systematic review literature highlighted that (i) logic models were infrequently used as a review tool and that the extent of use is likely to reflect the conventions of different disciplines; and (ii) where logic models were used, they were often used in a very limited way to represent the intervention pictorially. Often, they did not ostensibly appear to be used as tools integral to the review. There remains the possibility that some of the reviews and protocols featured earlier simply did not report the extent to which they used the logic model, although given that this is both a tool for thinking conceptually and a communication tool, it could be expected that the logic model would be referred to and referenced at different points in the review process. Logic models can be useful review tools, although the limited scope of use described in the literature could suggest that they are in danger of becoming a box-ticking exercise included in reviews and protocols rather than methodological aids in their own right.

### Terminology is important: Logic models and theories of change

We earlier stated that ‘theories of change’ and ‘logic models’ were used interchangeably by reviewers, largely dependent of the discipline in which they are conducted. However, outside the systematic review literature, a distinction often exists. Theories of change are often used to denote complex interventions where there is a need to identify the assumptions of how and why sometimes disparate elements of large interventions may effect change; they are also used in cases for less complex interventions where assumptions of how and why program components effect change are pre-specified. Theories of change can also be used to depict how entirely different interventions can lead to the same set of outcomes. Logic models on the other hand are used to outline program components and check whether they are plausible in relation to the outcomes; they do not necessitate the underlying assumptions to be stated [11, 75]. This distinction fits in well with the different stages of a systematic review. A logic model provides a sequential depiction of the components of interventions and their outcomes, but not necessarily the preconditions that are needed to achieve these outcomes, or the relative magnitude of different components. Given that few of the programme theory tools that are used in current protocols and reviews are derived or build upon existing tools, for most systematic reviews that do not constitute whole system reviews of complex intervention strategies, or for reviews that are not testing a pre-existing theory of change, developing a Logic Model initially may be most appropriate. This assertion does not mean that systematic reviews should be atheoretical or ‘theory-lite’, and different possible conceptual frameworks can be included in Logic Models. However, the selection of a single conceptual framework upfront, as is implicitly the case when developing a Theory of Change, may not represent the diversity of disciplines that reviewers are likely to encounter. Except in the cases outlined earlier around highly complex/systems based interventions (mainly restricted to development studies literature), theories of change are causal models that are most suitable when developed through the evidence gathered in the review process itself.

### Logic models can evolve into theories of change

Once a review has identified the factors that are associated with different outcomes, their magnitude, and the underlying causal pathways linking intervention components with different outcomes, this evidence can in some cases be used to update a logic model and construct a theory of change. We can observe examples in the literature where review evidence has been synthesised to map out the direction and magnitude of evidence in the literature (see [8], although in this case, the resulting model was described as a ‘Logic Model’ and not a ‘Theory of Change’), and this serves as a good model for all reviews. Programme theory can effectively be used to represent the change in understanding developed as a result of the review, and even in some cases the learning acquired during a review, although this is not the case for all reviews and there may be some where this approach is unsuitable or is otherwise not possible. A logic model can be viewed iteratively as a preliminary for constructing a theory of change at the end of the review, which in turn forms a useful tool to communicate the findings of the review. However, some reviewers may find little to update in the logic model in terms of the theory of the intervention or may otherwise find that the evidence around the outcomes and process of the intervention is unclear among the literature as it stands. There may also be occasions where reporting conventions for disciplines or review groups may preclude updating the logic model on the basis of the findings of the review.

### Programme theory should not be developed in isolation

In our exploration of health-based and international development reviews, we observed just one example where the reviewers described a Logic Model as having been developed through consensus within the review team [24]. Other examples are found in the literature, where logic models or theories of change have been developed with stakeholders, for example Baxter and colleagues [8; p3] record that ‘following development of a draft model we sought feedback from stakeholders regarding the clarity of representation of the findings, and potential uses’. These examples are clearly in the minority in the systematic review literature, although most programme theory described in the evaluation literature is clear that models should be developed through a series of iterations taking into account the views of different stakeholders [11]. While some of this effect may be due to reporting, as it is likely that at least some of the models included in Tables 1 and 2 were developed having reached a consensus, it is nevertheless important to highlight that a more collaborative approach to developing models could bring benefits to the review itself. Given that systematic review teams are often interdisciplinary in nature, and can be engaging with literature that is also interdisciplinary, programme theory should reflect the expertise and experience of all team members as well as that of external stakeholders if appropriate. Programme theory is also used as a shorthand communication tool, and the process of developing a working theoretical model within a team can help to simplify the conceptual model into a format that is understandable within review teams, but which can also be used to involve external stakeholders, as is often necessary in the review process [70].

### A logic model should be used as an Early Warning System

Logic models have their original purpose as a planning and communication tool in the evaluation literature. However in systematic reviews, they can also provide the first test of the underlying conceptual model of the review. If a review question cannot be represented in a Logic Model (or Theory of Change in the case of highly complex issues), this can provide a signal that the question itself may be too broad or needs further refining. It may be that a series of Logic Models may better represent the underlying concepts and the overall research question driving the review, and this may also reflect a need to undertake a series of reviews rather than a single review, particularly where the resources available may be more limited [73]. Alternatively, as is often the case with complex systems-based interventions (as encountered in many reviews of international development initiatives published on the 3ie database), the intervention may be based on a number of components, which could be represented individually through logic models, the mechanisms of which are relatively well-established and understood, and a theory of change may better represent the intervention. The tool can also help the reviewer to assess the degree to which the review may focus on collecting evidence around particular pathways of outcomes, and the potential contribution the review can make to the field, helping to establish whether the scope of the review is broad and deep (as might be the ideal in the presence of sufficient resource), or narrower and more limited in scope and depth [73]. This can also help to manage the expectations of stakeholders at the outset. Logic models can be used as the basis for developing a systematic review protocol, and should be considered living documents, subject to several iterations during the process of developing a protocol as the focus of the review is clarified. They can both guide and reflect the review scope and focus during the preparation of a review protocol.

### There is no set format for a Logic Model (or Theory of Change), but there are key components that should be considered

Most Logic Models at a minimum, depict the elements included in the PICO/T/S framework (patient/problem/population; intervention; comparison/control/comparator; outcomes and time/setting) [76]. However, a logic model represents a causal chain of events resulting from an intervention (or from exposure, membership of a group or other ‘treatment’); therefore it is necessary to consider how outcomes may precede or succeed one another sequentially. Dividing outcomes into distal (from the intervention), intermediate or proximal categories is a strategy that is often used to help identify sets of pre-existing conditions or events needed in order to achieve a given outcome. The result is a causal chain of events including outputs and outcomes that represent the pre-conditions that are hypothesised to lead to distal outcomes. Outcomes are only achieved once different sets of outputs are met; these may represent milestones of the intervention but are not necessarily success criteria of the intervention in themselves (for example in Fig 3). In the case of reviews of observational studies, the notion of outputs (and even interventions and intervention components) may be less relevant, but may instead be better represented by ‘causes’ and potential ‘intervention points’ [71], that are also structured sequentially to indicate which are identified as necessary pre-conditions for later events in the causal chain.

Many of the elements described above refer to the role of the intervention (or condition) in changing the outcomes for an individual (or other study unit), which can also be referred to as a theory of change; the elements of the causal chain that reflect the intervention and its modifiable elements are known as the theory of action [11, 77]. Within the theory of action, the modifiable components of the intervention needed to achieve later outputs and outcomes, such as the study design, resources, and process-level factors such as quality and adherence are usually included. Other modifiable elements, including population or group-level moderators can also be included, and even the underlying conceptual theories that may support different interventions may be included as potential modifiers. Finally, it some of the contextual factors that may reflect the environments in which interventions take place can also be represented. Within our example in Fig 3, these include the school-level factors such as intake of the school and its local neighbourhood as well as broader health service factors and local health policies. For some reviews and studies, the influence of these contextual factors may themselves be the focus of the review.

## Summary and Conclusions

In the past, whether justified or not, a critique often levelled at systematic reviews has been the absence of theory when classifying intervention components [78]. The absence of theory in reviews has transparent negative consequences in terms of the robustness of the findings, the applicability and validity of recommendations, and in the absence of mechanistic theories of why interventions work, limits on the generalisability of the findings. A number of systematic reviewers are beginning to address this critique directly through considering the methodological options available when reviewing theories [69, 78, 79], while others have gone further through exploring the role that differences in taxonomies of theory hold in explaining effect sizes [78, 80, 81]. Nevertheless, despite the benefits of, and need for, using theory to guide secondary data analysis, reviewers may be confronted by several situations where the conceptualisation of the theoretical framework itself may be problematic. Such instances include those where there may be little available detail on the theories underlying interventions, or competing theories or disciplinary differences in the articulation of theories for the same interventions exist (requiring synthesis) [79]; or a review topic may necessitate the grouping and synthesis of very different interventions to address a particular research question; or more fundamentally, where there is a need to consider alternative definitions, determinants and outcomes of interventions that goes beyond representing these within ‘black boxes’. In common with others before us [7, 8], in this paper we view a logic model as a tool to help reviewers to overcome these challenges and meet these needs through providing a framework for ‘thinking’ conceptually.

Much of this paper examines the application of a logic model to ‘interventionist’ systematic reviews, and we have not directly considered their use in systematic reviews of observational phenomena. Certainly, while some of the terminology would need to change to reflect the absence of ‘outputs’ and ‘resources’, the benefits to the review process would remain. For some, this idea may simply be too close that of a graphical depiction of a conceptual framework. However, the logic model is distinct in that it represents only part of a conceptual framework–it does not definitively represent a single ideological perspective or epistemological stance, and the accompanying assumptions. Arguably, a theory of change often does attempt to represent an epistemological framework, and this is why we view a distinction between both tools. As the goal of a systematic review is uncover the strength of evidence and the likely mechanisms underlying how different parts of a causal pathway relate to one another, then the evidence can be synthesised into a theory of change; and we maintain the emphasis on this being a ‘theory’, to be investigated and tested across different groups and across different geographies of time and space.

In investigating the use of logic models, we found that among the comparatively small number of reviewers who used a theory of change or logic model, many described a limited role as well as role intrinsic to the beginning of the review and not as a tool to communicate review findings. A worked through example may help expand the use as will making their use a formal requirement, but the formation of guidelines will help make sure where they are used, they’re used to greater effect. A recommendation of this paper is for greater guidance to be prepared around how programme theory could and should be used in systematic reviewing, incorporating the elements raised here and others. Much of this paper is concerned with the benefits that logic models can bring to reviewers as a pragmatic tool in carrying out the review, as a tool to help strengthen the quality of reviews, and perhaps most importantly as a communication tool to disseminate the findings to reviewers and trialists within academic communities and beyond to policy-makers and funders. With respect to this last purpose in particular, improving the way in which logic models are used in reviews can only serve to increase the impact that systematic reviews can have in shaping policy and influencing practice in healthcare and beyond.

## Supporting Information

S1 Checklist PRISMA Checklist. (DOCX)

S1 Flowchart PRISMA Flowchart. (DOCX)

S1 Table Data Coding Template. (DOCX)
