# Supplementary material for: Developing and Optimising the Use of Logic Models in Systematic Reviews: Exploring Practice and Good Practice in the Use of Programme Theory in Reviews
Source: PLoS One. 2015 Nov 17;10(11):e0142187. doi: 10.1371/journal.pone.0142187 (PMC4648510; doi:10.1371/journal.pone.0142187)
Supplement: S1 Flowchart — (DOCX) [file pone.0142187.s002.docx]

**PRISMA Flowchart statement:**

The study includes all 24 sources on the 3ie database that included mention of theory of change or logical model in the text, published in 2013 (of a possible 53 records published over this period).

The study includes all 15 sources on the Cochrane Library (Cochrane Reviews only) that included mention of theory of change or logical model in the text, published between September 2013 and September 2014 (of a possible 1,533 records published over this period).

Given the small number of studies and the focus of the present study in exploring the use of a specific methodological tool, all studies that met the criteria were included and their use of logic models or theories of change examined further using the coding template (S3). A PRISMA flowchart does not apply to the current study; the detail above enables replication of how studies were selected into the review.
