# Supplementary material for: Developing and Optimising the Use of Logic Models in Systematic Reviews: Exploring Practice and Good Practice in the Use of Programme Theory in Reviews
Source: PLoS One. 2015 Nov 17;10(11):e0142187. doi: 10.1371/journal.pone.0142187 (PMC4648510; doi:10.1371/journal.pone.0142187)
Supplement: S1 Table — (DOCX) [file pone.0142187.s003.docx]

| Type of publication | Protocol or Report | Indicate whether publication is a protocol or review |
| --- | --- | --- |
| Type of program theory tool | Logic Model or Theory of Change | Indicate whether tool used is logic model or theory of change |
| 1.  Review initiation |  | Indicate whether reported that tool is used to communicate aims of review in engaging with stakeholder or involving/recruiting different team members or obtaining funding. |
| 2. Review Question and Methodology | Used to describe how the intervention might work | Indicate whether reported that tool is used to communicate how the intervention may work |
|  | Developed from existing theories of change/logic models? | Indicate whether reported that tool is based on, or adapted from, exiting tools |
| 3.  Search strategy | Used to guide type of intervention (selection criteria) | Indicate whether reported that tool is used to make decisions on the inclusion criteria for studies in the review |
| 4. Description of study characteristics |  | Indicate whether reported that tool is used to make decisions on coding information on study characteristics |
| 5. Synthesis | Used to guide sub-group analyses | Indicate whether reported that tool is used to justify decisions on sub-group analyses |
|  | Logic Model used to structure qualitative synthesis | Indicate whether reported that tool is used to plan qualitative analysis (e.g. framework synthesis) |
|  | Used to synthesise qualitative and quantitative evidence | Indicate whether reported that tool is used to justify decisions on sub-group analyses |
| 6. Quality and relevance assessment |  | Indicate whether reported that tool is used as reference point in choosing quality assessment tools |
| 7. Using reviews (communication) | Did or plan to revise at the end of the review | Indicate whether reported that tool would be revised based on the review findings; indicate whether revised tool would be included in dissemination plans |
| Project Management | Referred to how Logic Model might be used structure the review | Indicate whether reported that tool is used as basis for planning review stages sequentially or used to communicate progress during the review |
| Additional considerations | Described as being constructed through consensus building | Indicate whether reported that tool is based on shared consensus across the team or across stakeholders |
